# Cardiac T2-mapping using a fast gradient echo spin echo sequence - first in vitro and in vivo experience

**DOI:** 10.1186/s12968-015-0177-2

**Published:** 2015-08-01

**Authors:** Bettina Baeßler, Frank Schaarschmidt, Christian Stehning, Bernhard Schnackenburg, David Maintz, Alexander C. Bunck

**Affiliations:** Department of Radiology, University Hospital of Cologne, Kerpener Str. 62, D-50937 Cologne, Germany; Institute of Biostatistics, Faculty of Natural Sciences, Leibniz Universität Hannover, Hannover, Germany; Philips Research, Hamburg, Germany; Philips, Healthcare Germany, Hamburg, Germany

**Keywords:** Cardiovascular magnetic resonance, T2-mapping, Parametric imaging

## Abstract

**Background:**

The aim of this study was the evaluation of a fast Gradient Spin Echo Technique (GraSE) for cardiac T2-mapping, combining a robust estimation of T2 relaxation times with short acquisition times. The sequence was compared against two previously introduced T2-mapping techniques in a phantom and *in vivo*.

**Methods:**

Phantom experiments were performed at 1.5 T using a commercially available cylindrical gel phantom. Three different T2-mapping techniques were compared: a Multi Echo Spin Echo (MESE; serving as a reference), a T2-prepared balanced Steady State Free Precession (T2prep) and a Gradient Spin Echo sequence. For the subsequent *in vivo* study, 12 healthy volunteers were examined on a clinical 1.5 T scanner. The three T2-mapping sequences were performed at three short-axis slices. Global myocardial T2 relaxation times were calculated and statistical analysis was performed. For assessment of pixel-by-pixel homogeneity, the number of segments showing an inhomogeneous T2 value distribution, as defined by a pixel SD exceeding 20 % of the corresponding observed T2 time, was counted.

**Results:**

Phantom experiments showed a greater difference of measured T2 values between T2prep and MESE than between GraSE and MESE, especially for species with low T1 values. Both, GraSE and T2prep resulted in an overestimation of T2 times compared to MESE. *In vivo*, significant differences between mean T2 times were observed. In general, T2prep resulted in lowest (52.4 ± 2.8 ms) and GraSE in highest T2 estimates (59.3 ± 4.0 ms). Analysis of pixel-by-pixel homogeneity revealed the least number of segments with inhomogeneous T2 distribution for GraSE-derived T2 maps.

**Conclusions:**

The GraSE sequence is a fast and robust sequence, combining advantages of both MESE and T2prep techniques, which promises to enable improved clinical applicability of T2-mapping in the future. Our study revealed significant differences of derived mean T2 values when applying different sequence designs. Therefore, a systematic comparison of different cardiac T2-mapping sequences and the establishment of dedicated reference values should be the goal of future studies.

## Background

Alterations of myocardial T2 relaxation times can be induced by a variety of diseases, including edema [1–3] and iron overload [4, 5]. Quantitative tissue characterization using T2-mapping is emerging as an important cardiovascular magnetic resonance (CMR) method, overcoming some of the known limitations of qualitative T2-weighted imaging [6–9]. Accordingly, it may lead to a more objective image interpretation and allow for a more sensitive detection of either diffuse or subtle focal changes in myocardial T2 relaxation times.

T2 maps are obtained by collecting multiple images with different T2-weighting, providing multiple points along the T2 decay curve for fitting of an exponential signal decay model [10, 11]. Several T2-mapping methods have been described, employing either fast spin-echo [12] techniques with multiple echo times, or T2-prepared (T2prep) steady-state free-precession (SSFP) techniques [13, 14] with multiple T2 contrast preparations.

The Multi Echo Spin Echo (MESE) technique is widely accepted as the reference method for T2-mapping, but it involves long scan durations and may yield artifacts - e.g. induced by motion and flow, or incomplete blood suppression. In contrast, the T2prep technique in concert with a balanced SSFP image readout is inherently flow-insensitive and sufficiently fast to be acquired in a single breathhold. Thus, it allows coverage of the entire myocardium at a reasonable expenditure of scan time and is therefore more suitable for routine clinical use. However, compared to the MESE technique, the number of echoes acquired by the T2prep sequence is considerable lower providing only a limited number of data points along the T2 decay curve, which may compromise accuracy and limit its use to a narrow range of T2 species.

In the current study we evaluate a T2-mapping sequence using a Gradient Echo Spin Echo (GraSE) technique [15], which provides a sufficient number of echoes comparable to the MESE technique, while offering scan times which are sufficiently short to be performed in a single breath-hold. In our study, the sequence is evaluated in a phantom as well as in a sample of healthy volunteers and compared against the T2prep and MESE sequences.

## Methods

### Phantom experiments

Phantom experiments were performed at 1.5 T (Achieva 1.5 T, Philips Medical Systems, Best, The Netherlands) using a commercially available cylindrical gel phantom (Eurospin test object TO5, Diagnostic Sonar, Livingston, UK) comprising 12 samples with T1 ranging from 185 ms to 1183 ms and T2 ranging from 50 ms to 160 ms, respectively. Identical imaging parameters as in the subsequent *in vivo* study were used (see below). For measuring T1, a Modified Look Locker Inversion Recovery sequence (MOLLI) was used. Typical imaging parameters were: TR/TE = 2.3/1.15 ms, FA 35 °, parallel imaging (SENSE = 2.0), eight single shot balanced SSFP readout trains (inversion, three readouts in consecutive RR intervals, three pause intervals to allow magnetization recovery, re-inversion, five consecutive read-outs).

### Study population

12 healthy volunteers were enrolled into the study (6 men/6 women (Table 1). Inclusion criteria for volunteers were: i) uneventful medical history, ii) no symptoms of inflammation, iii) absence of any symptoms indicating cardiovascular dysfunction, iv) normal cardiac dimensions and function proved by cine CMR. We discouraged alcohol intake and high-intensity sportive activities 24 h before the scans to avoid inflammatory reaction [16, 17] and activity-dependent changes in T2 [6]. Volunteers with history of inflammatory disease including common cold virus in the last four weeks before the scans were excluded from the study [18].

**Table 1:** Characteristics of the volunteers

Parameter	Result
Number	12
Females/Males	6/6
Age [years]	33 ± 11
Height [cm]	177 ± 10
Weight [kg]	74 ± 14
Body mass index [kg/m^2^]	23 ± 3
Body surface area [m^2^]	1.9 ± 0.2
Heart rate [min^−1^]	61 ± 11
LV enddiastolic volume [ml]	166 ± 39
LV enddiastolic volume index [ml/cm]	0.9 ± 0.2
LV ejection fraction [%]	60 ± 5
LV mass [mg]	97 ± 31
LV mass index [mg/cm]	0.5 ± 0.1

*LV* left ventricle

The study was approved by the local ethical committee and written informed consent given by all volunteers. All experiments were performed in compliance with the Helsinki Declaration.

### CMR examination

Each subject had CMR on a clinical 1.5 T scanner (Achieva 1.5 T, Philips Medical Systems, Best, The Netherlands) using a 5-channel cardiac phased array receiver coil and a 4-lead vectorcardiogram.

#### Cine imaging

SSFP cine images were obtained during repeated breath-holds in two long axes and in a stack of short axes (SAX) covering the left ventricle (LV) to rule out wall motion abnormalities and allow for cardiac chamber quantification. Imaging parameters were: repetition time (TR) 2.8 ms, echo time (TE) 1.4 ms, flip angle (FA) 60 °, field of view (FOV) 343 × 380 mm^2^, matrix 256 × 256, slice thickness 8 mm, 50 cardiac phases.

#### T2-mapping

**Fig. 1:**
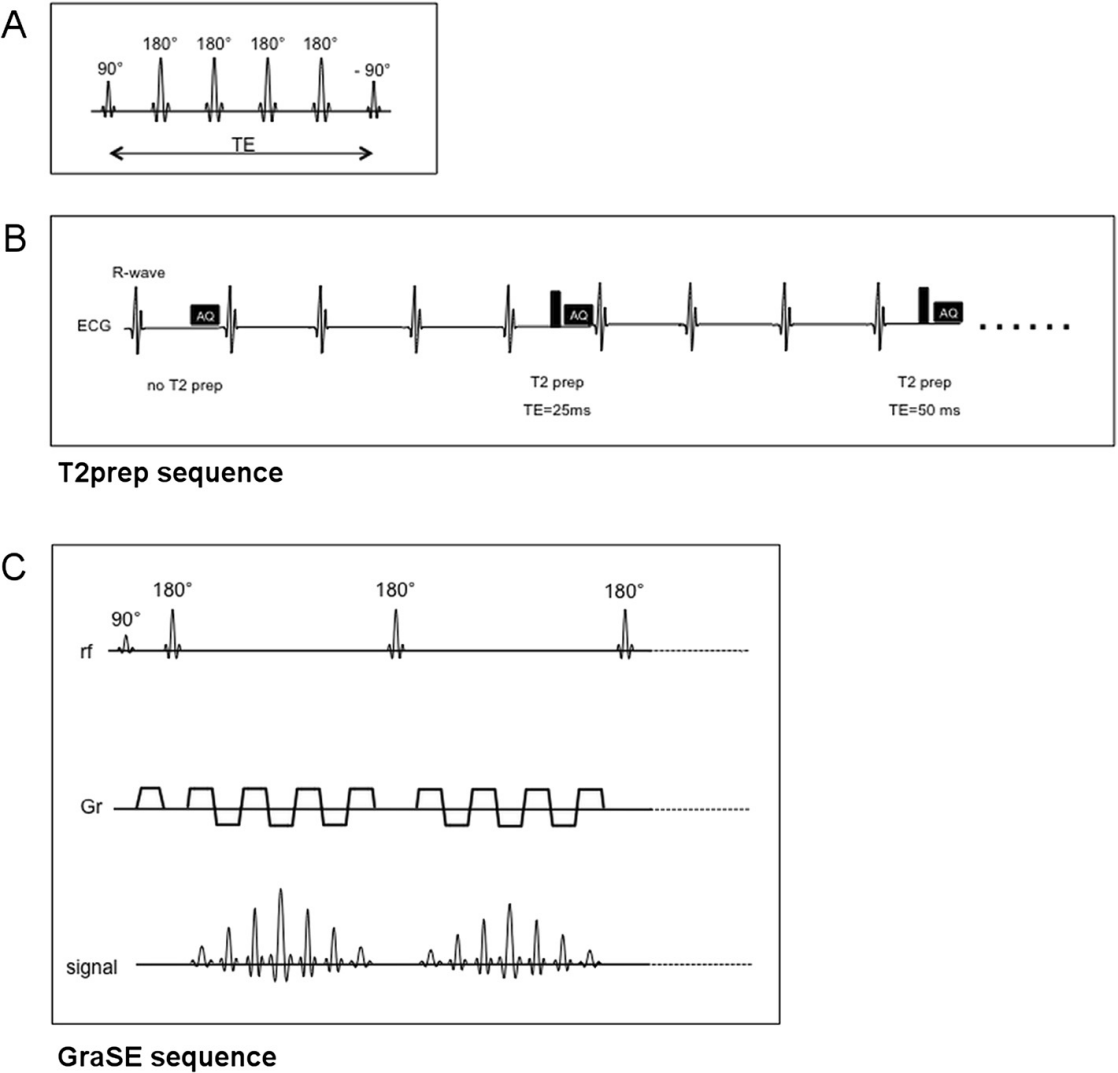
Schematic illustration of T2prep and GraSE sequences. **a** T2-preparation pulse scheme. **b** T2-mapping scheme [13, 14]: Single shot images with different T2preparation times with a gap of 3 RR intervals (T1 recovery). Example with 3 images. **c** GraSE Sequence scheme: a RF-refocused echo train is formed and seven gradient-recalled echoes are created during each RF echo. Example with two RF echoes. AQ - Acquisition of the image; RF - Radiofrequency

For T2-mapping, data were acquired in a basal, midventricular, and apical SAX plane using three different T2-mapping sequences: i) a T2-prepared single-shot balanced SSFP technique (T2prep; Fig. 1a, b) [9, 14], ii) a Gradient Spin Echo technique (GraSE; Fig. 1c) [15], and iii) a Multi Echo Spin Echo (MESE) technique that served as reference. For T2prep and GraSE, measurements were repeated once. The spatial resolution was equal for all methods (2 × 2 × 10 mm^3^). The three sequences were ECG triggered and had the following parameters: T2prep: TR/TE = 2.3/1.15 ms, FA 35 °, parallel imaging (SENSE = 1.6), TE’s of the T2prep pulse: 0, 25, 50, 75 ms and breath hold (scan duration about 12 s); GraSE: TR = 1 heartbeat, 9 echoes (TE_1_ = 15 ms, delta TE = 7.7 ms), FA 90 °, parallel imaging (SENSE = 2), EPI factor = 7, black blood prepulse and breath hold (scan duration about 14 s); MESE: TR = 1 heartbeat, 9 echoes (TE_1_ = 12 ms, delta TE = 5.8 ms), FA 90 °, parallel imaging (SENSE = 2), black blood prepulse and navigator gating (mean scan duration about 5 min). A pixel-wise myocardial T2-map was generated using a monoexponential fit [7, 9, 13] on the magnitude data using a maximum likelihood estimator (MLE), where a Rician noise distribution was assumed.

### CMR image analysis

#### LV chamber quantification

Image analysis was done using Philips Extended MR WorkSpace (version 2.6.3.4; Philips Medical Systems, Best, The Netherlands). SSFP cine images were visually evaluated in order to exclude wall motion abnormalities. LV enddiastolic and endsystolic volume and LV mass were measured by manually contouring the endocardial and epicardial borders of the SAX in systole and diastole using Simpson’s method and indexed according to the body surface area.

#### T2-mapping – quantitative assessment

**Fig. 2:**
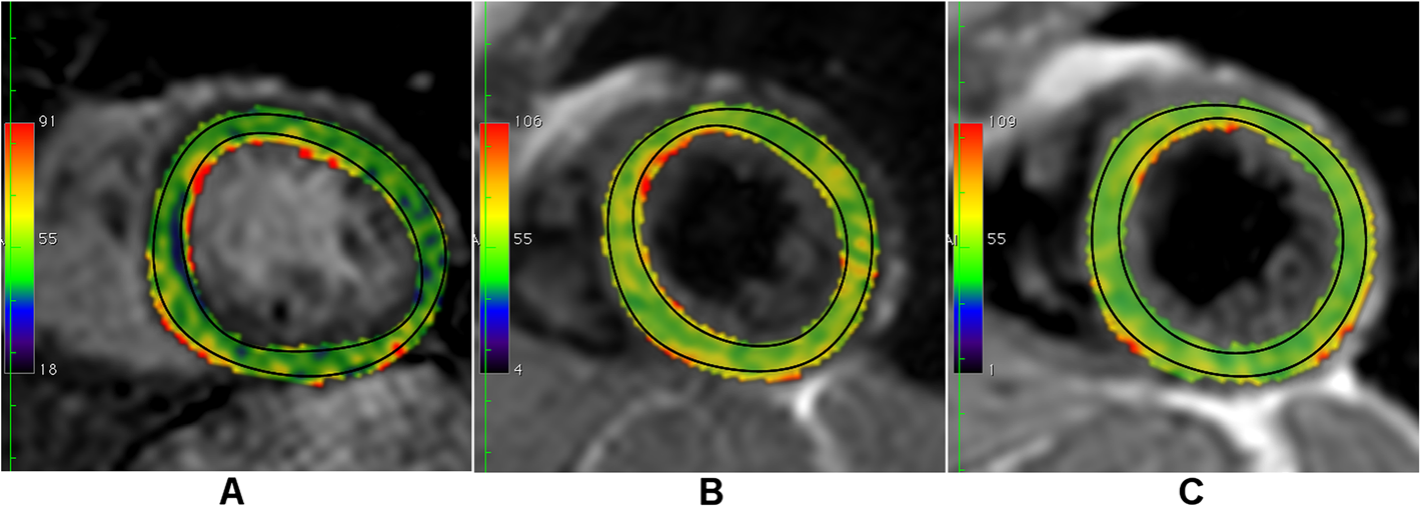
Exemplary T2-maps acquired with the three different T2-mapping sequences. Set of T2 maps in a midventricular slice of the same healthy volunteer, acquired with the three different sequences T2prep (**a**), GraSE (**b**) and MESE (**c**). The black regions of interest indicate those used for finale analysis of myocardial T2 in order to exclude partial volume artifacts at the endo- and epicardial borders of the left ventricle

T2 map reconstruction and image analysis was done with OsiriX viewer for Mac OS X (version 5.8.5, Pixmeo, Bernex, Switzerland). T2 maps were calculated with a dedicated plug-in written for the OsiriX software. Selected T2-maps for each sequence in one healthy volunteer are shown in Fig. 2. An endocardial and epicardial contour was drawn in one original image. The trabeculated layer and the epicardial border were left out. In doubtful cases, SSFP cine images were consulted. The contours were copied to the other images and aligned in each source image to correct for respiration-induced rigid body motion. The myocardial region of interest (ROI) was automatically segmented according to the AHA segment model [8, 19]. Results were calculated per segment and averaged over the whole myocardium.

#### *T2-mapping – segment-wise assessment* of pixel-by-pixel *homogeneity*

For assessment of pixel-by-pixel homogeneity in T2-maps the number of segments, in which the pixel SD exceeded 20 % of the mean T2 time of the corresponding segment, was counted. All statistical analyses were performed twice: i) without exclusion of any segment and ii) with exclusion of all segments demonstrating pixel-by-pixel inhomogeneity as defined above.

### Statistical analysis

Statistical analysis was performed in R 3.1.0 [20], using the packages nlme [21] and lme4 [22] for fitting mixed effect models [23], and the package multcomp [24] for multiple comparisons based on the fitted models.

In the phantom experiments, the paired differences of the observed T2 times of the T2prep and GraSE sequence vs. that of the MESE sequence were tested for significance using paired *T*-Test.

Linear mixed effects models (with random effect for subject and for scans within subjects) were used to account for repeated measurements within each subject, allowing for different residual variances in different sequences [21]. Tukey-type multiple comparisons [24] were performed to test for all pair-wise intraindividual differences between sequences.

For analysis of pixel-by-pixel homogeneity, observed proportions of segments in which the pixel-SD exceeded 20 % of the mean T2 time of the corresponding segment were analyzed in a generalized linear mixed model (logit-link, binomial assumption for the observed number of inhomogeneous segments) with a fixed effect for the sequences and random effects for subjects and repeated scans within subjects. The overall effect of sequences was assessed using a likelihood ratio test. Tukey-type comparisons between the three sequences (adjusted for multiple comparisons) were performed based on the model estimates [24].

## Results

### Phantom experiments

The results of the phantom experiments are shown in Table 2. Phantom experiments showed that significantly higher T2 values were measured using T2prep or GraSE, when compared to the MESE sequence (p < 0.001 for both). For GraSE, a smaller overestimation of T2 was observed in general when compared to T2prep (mean difference 6.4 ms), which was invariant to the T1 of the respective species. For T2prep, more pronounced deviations to the T2 values measured with MESE (mean difference 14.5 ms) were observed, particularly for species with short T1 (<500 ms).

**Table 2:** Results of the phantom experiments

	T1 MOLLI [ms]	T2 ref [ms]	T2 GraSE [ms]	T2 T2prep [ms]	Δ ref/GraSE [%]	Δ ref/T2prep [%]
1	313	67.7 ± 0.7	70.4 ± 1.9	95.1 ± 3.3	+4	+41
2	478	50.3 ± 0.8	53.6 ± 2.4	64.1 ± 3.0	+7	+27
3	606	86.9 ± 1.1	90.5 ± 2.2	101.2 ± 4.2	+4	+16
4	617	121.4 ± 1.6	127.7 ± 1.8	137.7 ± 6.2	+5	+13
5	793	105.8 ± 1.6	112.7 ± 3.3	113.4 ± 4.5	+6	+7
6	778	145.8 ± 2.7	159.3 ± 6.3	161.3 ± 10.4	+9	+11
7	941	123.7 ± 1.8	131.3 ± 7.2	137.2 ± 6.9	+7	+11
8	1046	196.4 ± 4.6	203.7 ± 3.8	211.4 ± 7.2	+4	+8
9	1072	148.7 ± 3.2	158.6 ± 4.4	158.5 ± 6.6	+7	+7
10	1234	190.3 ± 5.8	203.2 ± 7.7	210.7 ± 18.3	+7	+11
11	1345	166.9 ± 3.6	182.0 ± 6.0	186.5 ± 13.6	+9	+12
12	1556	161.3 ± 5.7	173.5 ± 9.2	177.3 ± 15.2	+8	+10

Δ ref in % indicates the percental difference from the reference standard (MESE)

*MOLLI* Modified Look Locker Inversion Recovery; Values are given ± standard deviation

### *In vivo* experiments

**Fig. 3:**
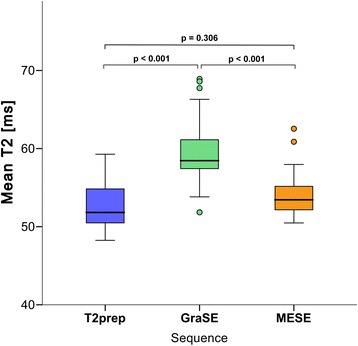
Differences between the three T2-mapping sequences. Box-Whisker plots representing the differences of global myocardial T2 times [ms] between the three T2-mapping sequences. The centerline in each box represents the median, whereas the lower and upper limits of each box represent the 25th and 75th percentiles, respectively. Whiskers extend to the most extreme observations within median ±1.5*IQR. Observations outside median ± 1.5*IQR are shown as dots. IQR - inter-quartile-range

*In vivo* global myocardial T2 relaxation times for each sequence are summarized in Table 3. GraSE yielded highest and T2prep lowest T2 estimates (Fig. 3). T2 values obtained with GraSE were significantly higher than those obtained with T2prep (59.3 ± 4.0 vs. 52.4 ± 2.8 ms, p < 0.001) and MESE (59.3 ± 4.0 vs. 53.8 ± 2.5 ms, p < 0.001). Comparing T2prep and MESE_,_ T2 values differed slightly, but differences proved to be not significant at the 5 % level (52.4 ± 2.8 vs. 53.8 ± 2.5 ms, p = 0.306).

**Table 3:** Global myocardial T2 relaxation times [ms] for different sequences

Sequence	T2 [ms]	SD	CI (2.5 − 97.5 %)
T2prep	52.4	2.8	50.6 − 54.2
GraSE	59.3	4.0	56.8 − 61.8
MESE	53.8	2.5	52.2 − 55.4

*SD* standard deviation between subjects, *CI* confidence interval for the mean T2 time

**Fig. 4:**
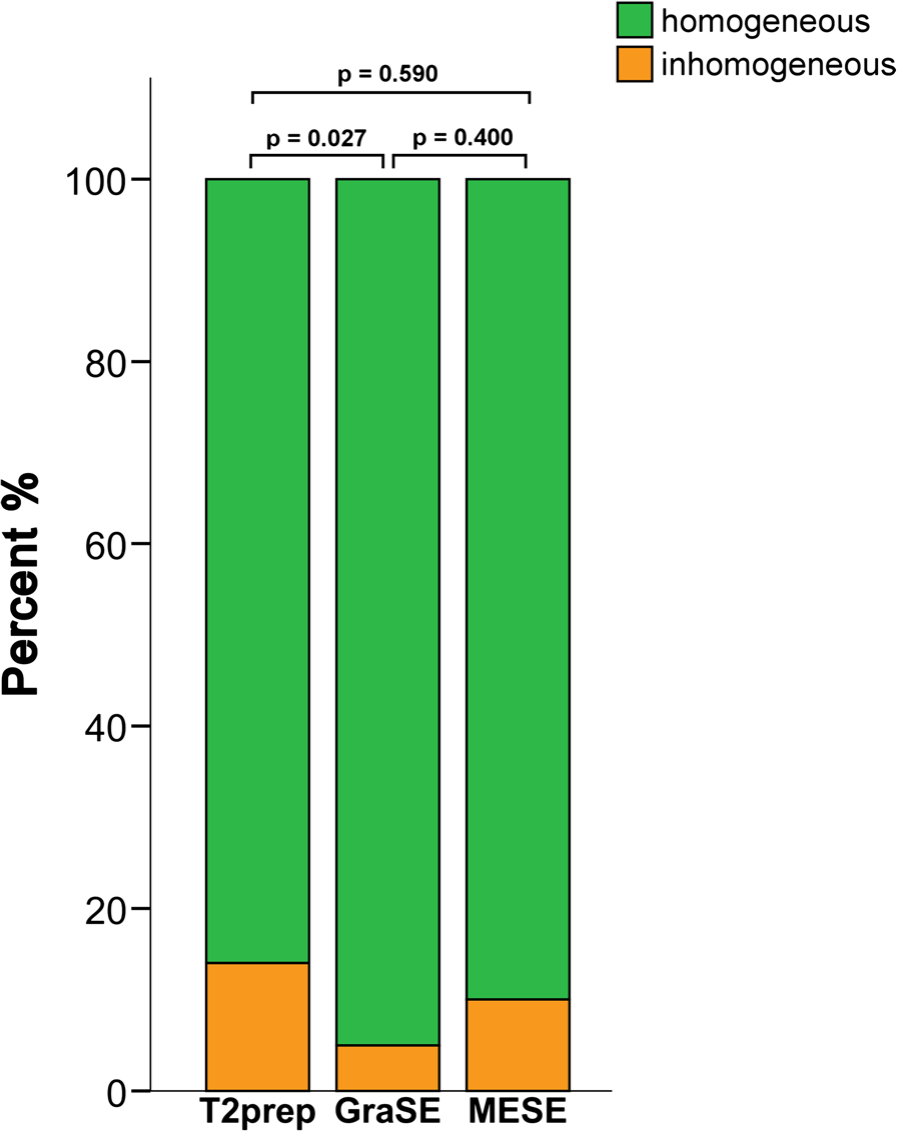
Assessment of segmental pixel-by-pixel homogeneity. Percentage of segments demonstrating a pixel SD exceeding 20 % of the corresponding observed T2 times (orange; inhomogeneous) and not exceeding 20 % of the corresponding observed T2 times (green, homogeneous), separately for each sequence

Analysis of pixel-by-pixel homogeneity per segment revealed a variability of T2 values by a standard deviation of more than 20 % of the mean T2 time of the corresponding segment in 8 % of all analyzed myocardial segments. The highest number of segments meeting the definition of inhomogeneity was found for T2prep (14 %), followed by MESE (10 %) (Fig. 4). Conversely, GraSE showed only a relatively small number of segments affected by inhomogeneity (5 %). The difference in the number of inhomogeneous segments between GraSE and T2prep proved to be significant at the 5 % level (p = 0.027).

Inclusion or exclusion of inhomogeneous myocardial segments did not yield significant changes of the mean T2 times, nor did it exclude extreme values/outliers. Due to these observations, no data were excluded in the statistical analyses.

## Discussion

In the present study, we introduced a fast T2-mapping sequence (GraSE) and compared it against a T2prep and a MESE sequence in a phantom and *in vivo*. While T2 is an inherent tissue property, previous studies have provided evidence that different T2-mapping sequence designs may introduce variations in the measured T2 values [7, 13]. Accordingly, our study revealed significant variations for the derived T2 values depending on the sequence used.

In our phantom study, both the T2prep and the GraSE sequence yielded higher T2 values for the sample probes compared to the MESE sequence, which served as our reference. This overestimation was most pronounced for the T2prep sequence and is in good agreement with previous reports by Giri et al., where a similar overestimation of T2 values by the T2prep sequence was found in phantoms [13]. In addition to previous studies [13], we observed a T1 shine through effect in terms of increasing T2 differences with respect to the reference with decreasing T1 of the respective probe in the phantom study. A pronounced overestimation of T2 was observed predominantly in phantom samples with short T1 (<500 ms), which had not been reported earlier, presumably due to the narrow range of species included in those studies. Therefore, the T2prep sequence may not be suitable for post-contrast conditions with reduced myocardial T1, which is an aspect that should be addressed in a further study in an appropriate patient cohort.

*In vivo*, a minor trend towards an underestimation of T2 was observed using the T2prep sequence in healthy myocardium with T2 < < T1, although the myocardial T2 values obtained with T2prep were in good agreement with previously reported values in the literature [7, 13, 25, 26]. This renders the T2prep sequence well suited for the assessment of T2 in normal myocardium in native condition.

Conversely, a slight overestimation of T2 values was observed for the GraSE when compared with the MESE sequence. This small deviation was somewhat more pronounced in our in-vivo than in our phantom measurements (10 % vs. 6 %), which is most likely due to the longer echo-spaces of GraSe (7.7. ms vs. 5.8 ms) necessary to fit the Echo Planar Imaging (EPI) readouts, thereby making it more susceptible to motional blurring and through plane motion.

Using a black blood prepulse, GraSE might be more prone to bright-blood artifacts due to incomplete blood nulling compared to T2prep, which does not use a black blood prepulse. In our cohort of healthy volunteers, however, we rarely observed bright blood artifacts. Nevertheless, we used a post-processing method with manual ROI-definition that allowed us to exclude any partial volume or bright blood artifact from quantitative analysis. Therefore, we assume that bright blood artifacts – if present - did not influence the obtained T2 values in the present study. It should be kept in mind, however, that bright blood artifacts might be more prevalent in critically ill patients with a reduced ejection fraction. Therefore, in a future study, the comparison of GraSe and T2prep should be performed in a patient cohort in a future study to investigate image quality and the potential influence of artifacts like those caused by incomplete blood nulling on the derived T2 times.

When comparing interindividual variability of T2 values derived from the three sequences, standard deviation was marginally higher for the GraSE sequence. Conversely, homogeneity of pixel-by-pixel T2 values within a myocardial segment was highest for the T2 maps generated by the GraSE sequence. Compared to the T2prep sequence, nearly only a third the number of segments that showed pixel T2 values to vary by more than 20 % of the mean T2 time of the corresponding segment were found when using the GraSE sequence. As we do not expect the T2 values to vary significantly within a myocardial segment in a healthy volunteer, this higher homogeneity speaks in favor of a higher robustness of the T2 maps derived by the GraSE sequence when compared to the other mapping sequences.

The combination of a higher number of echoes acquired along the T2 decay curve (9 echoes for GraSE and MESE compared to 4 echoes for T2prep) [27], a lower sensitivity of the spin-echo based techniques (GraSE, MESE) to B0 inhomogeneities compared to SSFP-based techniques (T2prep) as well as a lower susceptibility to motion artifacts due to shorter acquisition times compared to the MESE may all contribute to this higher intra-individual consistency of T2 values derived by the GraSE sequence.

A higher intra-individual consistency of T2 values is of particular importance when it comes to the definition of objective cut-off values for the differentiation of focal edema from remote unaffected myocardium in pathologies with focal disease manifestation, like in myocarditis. As T2 times may vary between segments, we set the SD of pixel intensities in relation to the respective segmental T2 mean. In case of focal disease manifestation, we assume that disease will result in an increased inhomogeneity of T2 times. While overall T2 times are known to vary considerably between individuals [25], thus complicating the definition of commonly valid T2 thresholds to distinguish between health and disease, an intra-individually increased inhomogeneity may help to identify disease in the presence of normal, remote myocardium. Potential advantages of this approach in edema imaging on an individual per-patient basis should be the investigated in further studies.

### Study limitations

The results presented in our study are the first *in vivo* experiences for this newly introduced T2-mapping GraSE sequence at 1.5 T and we examined only a small number of healthy volunteers. Study limitations include the usual limitations for cardiac T2 imaging, i.e. the sensitivity to B1 inhomogeneity, the presence of stimulated echo pathways that cause the signal to decay with a mixture of T1 and T2 contributions, and the sensitivity to cardiac motion. As 3 T scanners are increasingly used for cardiac imaging, further studies are needed to investigate whether the identified advantages can equally be transferred to higher field strengths. Moreover, larger study cohorts are needed to establish reference values for the GraSE sequences and evaluate its potential clinical value in the detection of disease. Furthermore, the fast T2 mapping sequences (GraSE, T2prep) were optimized for patient comfort, i.e. short breathholds, rather than best possible T2 accuracy, and the same settings were used *in vivo* and in phantoms. The value of a potential individual edema detection by using the here employed pixel-by-pixel inhomogeneity should be the aim of further studies in an appropriate patient population. Moreover, the idea of intraindividual consistency of T2 times should be transferred to a larger context, e.g. by comparing mean T2 times on a segment- or slice-based approach. This should be done in a larger proband cohort to reach statistical significance.

## Conclusions

The GraSE sequence introduced in our study is a fast and robust sequence for myocardial T2-mapping, combining advantages of both MESE and T2prep techniques. By these means, this fast technique may facilitate the applicability of T2-mapping in clinical routine. Its improved intra-individual consistency of T2 values may be of particular value in the detection of acute focal myocardial disease, overcoming the known limitations of qualitative image assessment. Our study revealed significant differences of derived mean T2 values when applying different sequence designs underlining the necessity for dedicated reference values for each sequence.

## Abbreviations

AQ: Acquisition of the image
CI: Confidence interval
CMR: Cardiovascular magnetic resonance
FSE: Fast Spin Echo sequence
GraSE: Gradient Spin Echo sequence
IQR: Inter-quartile-range
LV: Left ventricle
MESE: Multi Echo Spin Echo sequence
MR: Magnetic resonance
RF: Radiofrequency
SAX: Short axis
SD: Standard deviation
SSFP: Steady-State Free-Precession
T: Tesla
T2prep: T2-prepared SSFP sequence
TSE: Turbo Spin Echo sequence
